# Supplementary material for: Acid–Base Chemistry of Short Hydrogen Bonds: A Tale of Schrödinger’s Cat in Glutamine-Derived Crystals
Source: J Phys Chem Lett. 2025 Aug 13;16(33):8588–95. doi: 10.1021/acs.jpclett.5c01499 (PMC12376103; doi:10.1021/acs.jpclett.5c01499)
Supplement: Supplementary file 1 [file jz5c01499_si_001.pdf]

Supporting Information: Acid base chemistry of short hydrogen bonds: a tale of Schrödinger's cat in Glutamine derived crystals

Muhammad Nawaz Qaisrani,[*,†,‡] Nandha Kumar,[¶,‡] Christian Dreßler,[†] Ralph Gebauer,[‡] and Ali Hassanali[*,‡]

† *Ilmenau University of Technology, Theoretical Solid State Physics*

Weimarer Straße 32, 98693 Ilmenau, Germany

‡ *ICTP - The Abdus Salam International Centre for Theoretical Physics*

Strada Costiera 11, 34151, Trieste, Italy

¶ *Department of Biomaterials (Prosthodontics), Saveetha Dental College and Hospitals,*

Chennai, Tamil Nadu 600077, India

E-mail: muhammad-nawaz.qaisrani@tu-ilmenau.de; ahassana@ictp.it

1. Crystal Structures of L-glutamine and L-pyro-amm

L-glutamine crystallizes in the orthorhombic $P2_12_12_1$ space group with four molecules in the unit cell.[1] These molecules are held together by an extended network of hydrogen bonds. Figure 1(a) presents the unit cell highlighting three representative hydrogen bonds (HB1–HB3). These hydrogen bonds span distances between 2.7 and 2.9,Å, which is typical for standard N–H···O hydrogen bonds and remain well-localized even under quantum condi-

tions. The nature and strength of these hydrogen bonds form a baseline for understanding deviations observed in the thermally derived phase.

Upon thermal incubation at 60,°C, L-glutamine undergoes cyclization, forming a new crystal composed of pyroglutamic acid, pyroglutamate, and ammonium ions.[2] The resulting L-pyro-amm structure also adopts the orthorhombic $P2_12_12_1$ symmetry. Figure 1(b) shows this new crystal configuration. Unlike L-glutamine, L-pyro-amm contains a chemically distinct short hydrogen bond (SHB) of approximately 2.5,Å formed between two carboxylate oxygen atoms. This SHB is embedded within a highly asymmetric local environment influenced by a nearby ammonium ion, which interacts with twelve different oxygen atoms, creating a complex hydrogen bonding network.

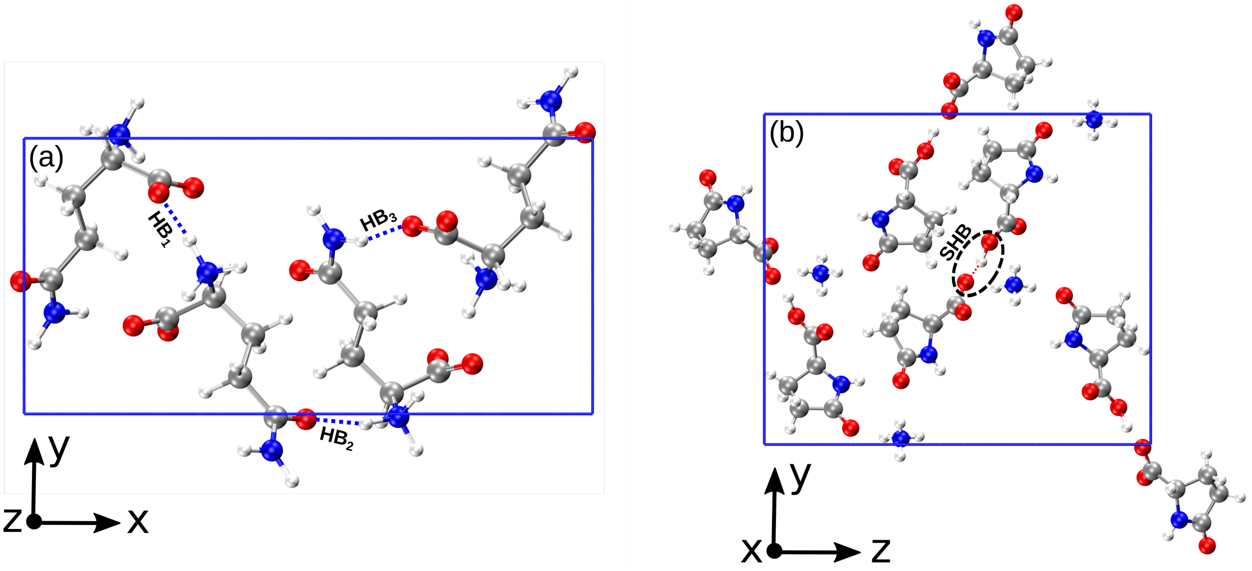

Figure 1: (a) Crystal structure of L-glutamine with hydrogen bonds HB1–HB3 (2.7–2.9 Å). (b) Crystal structure of L-pyro-amm highlighting the SHB and nearby ammonium ion.

2. Convergence of Path Integral Simulations

Ensuring convergence with respect to the number of beads used in path integral simulations is essential for quantitative reliability. We compared proton transfer coordinate distributions obtained using 6 and 8 beads within the PIGLET thermostat. Figure 2 confirms that the

6-bead simulation captures the key structural features of proton delocalization, with negligible deviations from the more computationally expensive 8-bead run. This validates our use of 6 beads in all production simulations.

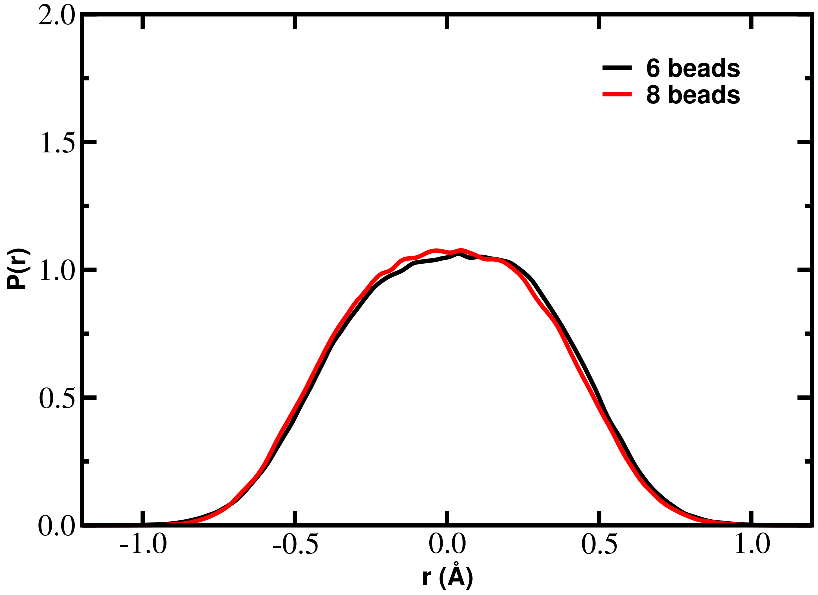

Figure 2: Proton transfer coordinate distributions for SHB with 6 and 8 beads in PIGLET simulations.

3. Proton Transfer Profiles in L-glutamine

To contextualize the unique behavior of the SHB in L-pyro-amm, we also examined proton transfer potentials in L-glutamine. As shown in Figure 3, even the shortest hydrogen bonds in L-glutamine remain single-welled with no barrier suppression, consistent with a conventional, localized proton. This further supports the conclusion that SHB symmetrization observed in L-pyro-amm is not merely a consequence of reduced bond length, but results from a combination of geometric constraint and local electrostatic modulation by the ammonium ion.

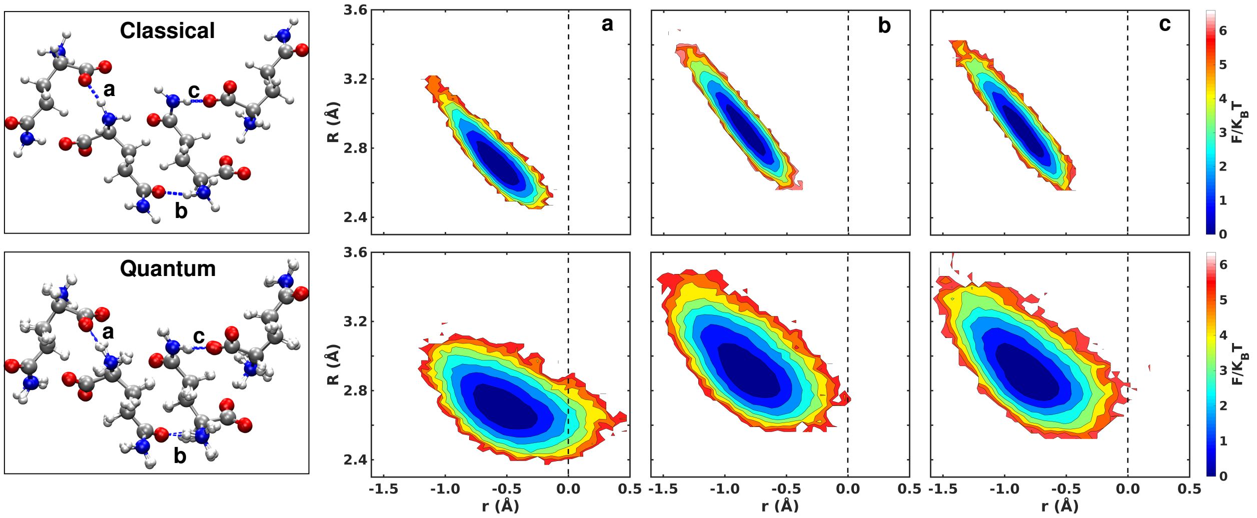

Figure 3: Free energy profiles of proton transfer coordinate for SHB in L-glutamine along its different hydrogen bonds, under classical and quantum simulations. No barrier suppression observed.

4. Wannier Center Distributions: SHBs vs. Normal Hydrogrn Bonds

To probe electronic structure changes induced by quantum fluctuations, we analyzed the distributions of maximally localized Wannier centers (WCs) associated with oxygen atoms in hydrogen bonds. Figure 4 shows the WC distributions for the SHB oxygen atom in L-pyro-amm (a same showin the main text), as well as for the hydrogen bonds formed between the ammonium ion and surrounding oxygen atoms. Classical simulations exhibit well-separated peaks corresponding to lone pairs and bonded electron pairs. In contrast, the quantum distributions are broadened, with partial overlap between lone pair and bonded WCs—indicating a delocalization of electron density and a transition from hydrogen bonding toward partial covalency.

For comparison, Figure 5 presents WC distributions for the hydrogen bonds in L-glutamine. Even under quantum conditions, these hydrogen bonds maintain a clear separation between lone pair and bonded WCs, demonstrating that the behavior observed for the SHB in L-pyro-amm is not universal, but rather dependent on specific geometric and electrostatic

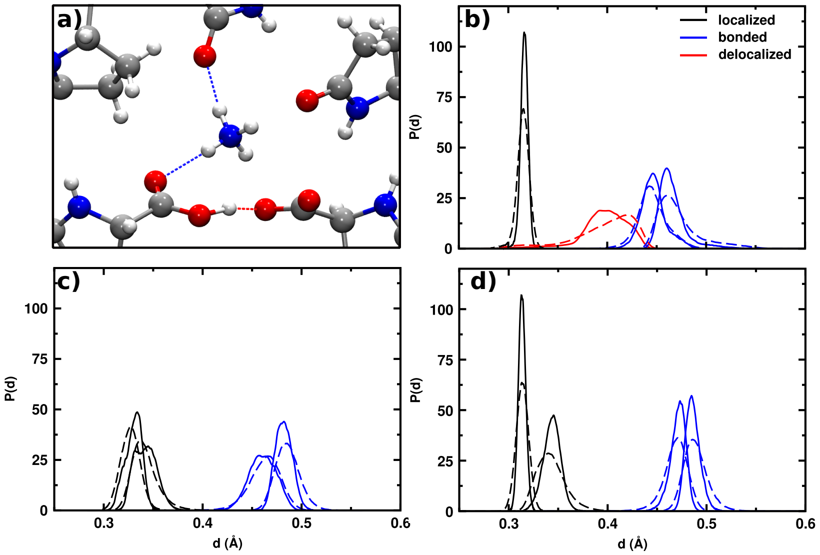

Figure 4: **(a)** Zoomed-in structural snapshot of L-pyro-amm showing representative hydrogen bonds formed between the ammonium ion and surrounding oxygen atoms (blue dashed lines), as well as the short hydrogen bond (SHB) highlighted with a red dashed line. **(b)** Classical (solid lines) and quantum (dashed lines) distributions of Wannier centers projected along the O–H bond axis for the SHB oxygen. **(c, d)** Wannier center distributions for the normal hydrogen bonds involving the ammonium ion, illustrating differences between classical and quantum conditions.

environments.

5. Structural Correlation Between Proton Transfer and Ion Vibrational Modes

Figure 6 shows joint probability distributions between the proton transfer coordinate (r) and selected ammonium rocking modes, defined as $d_{i,j} = d_i - d_j$, obtained from classical simulations. The modes shown here correspond to all distance pairs (i, j) for which the absolute value of the Pearson correlation coefficient is larger then 0.55. These distributions highlight the correlated fluctuations between proton position and low-frequency ammonium vibrational modes, consistent with the coupling mechanism discussed in the main text for the representative $d(6, 10)$ mode.

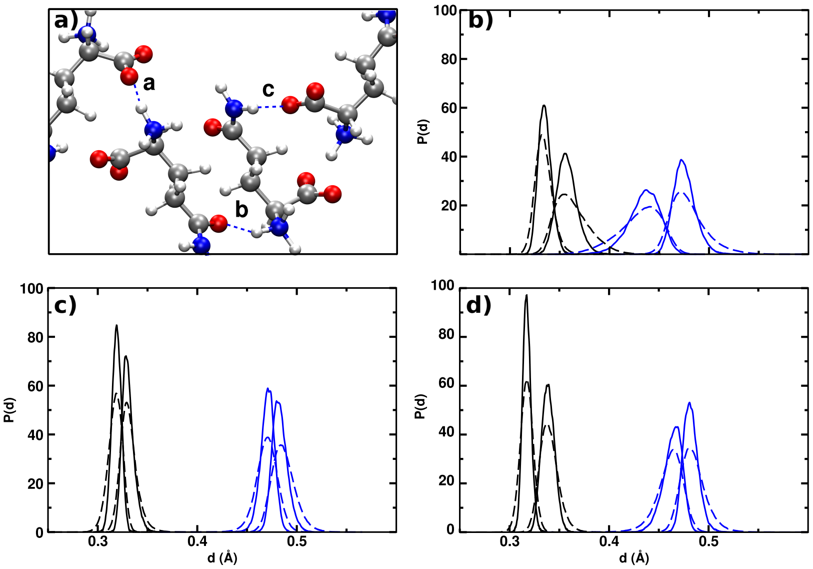

Figure 5: **(a)** Zoomed-in structural snapshot of L-glu showing its hydrogen bonds (a, b, and c) formed between terminal groups and between terminal and side-chain groups. **(b)** Distribution of Wannier centers for the terminal hydrogen bond, showing strong polarization compared to the hydrogen bonds involving side-chain groups (b and c), as shown in panels (c) and (d), respectively.

6. Benchmarks for AIMD Simulation Parameters

To validate the choice of basis set and plane-wave cutoff used in the path-integral molecular dynamics (PIMD) simulations, we performed benchmark calculations on 10 representative trajectory frames where a proton transfer event occurs along one of the short hydrogen bonds in the system. For each frame, single-point total energies were computed using the BLYP functional with D3 dispersion correction, across the following levels of theory:

- DZVP basis set with 300 Ry and 400 Ry electronic density cutoffs
- TZVP basis set with 300 Ry, 400 Ry, and 450 Ry electronic density cutoffs

Figure 7 shows the relative total energies computed at each level were compared, taking one frame as reference. The maximum deviation across all tested combinations was found to be less than 0.013 eV (13 meV). These results demonstrate that the DZVP basis set with a 300 Ry cutoff offers a reliable and computationally efficient choice for capturing the relative energetics relevant to proton transfer processes in this system. The use of more demanding

TZVP and higher cutoffs did not lead to significant improvements in relative energy accuracy, but would considerably increase the cost of ab initio PIMD simulations.

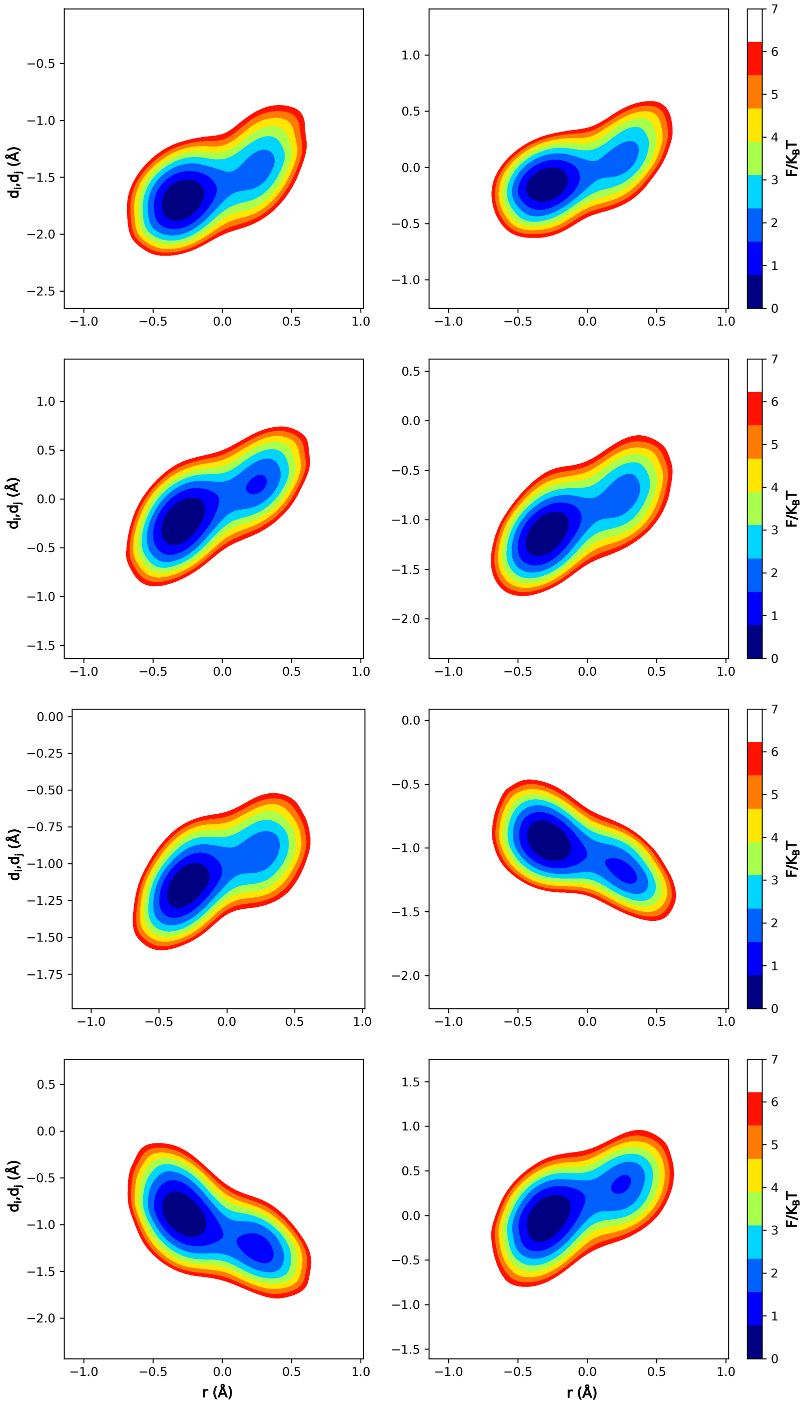

Figure 6: Joint probability distributions between the proton transfer coordinate (r) and ammonium rocking modes defined by the distance difference $d_{i,j} = d_i - d_j$, from classical simulations. The plotted modes are selected based on a Pearson correlation coefficient magnitude $|r| > 0.55$ with the PT coordinate.

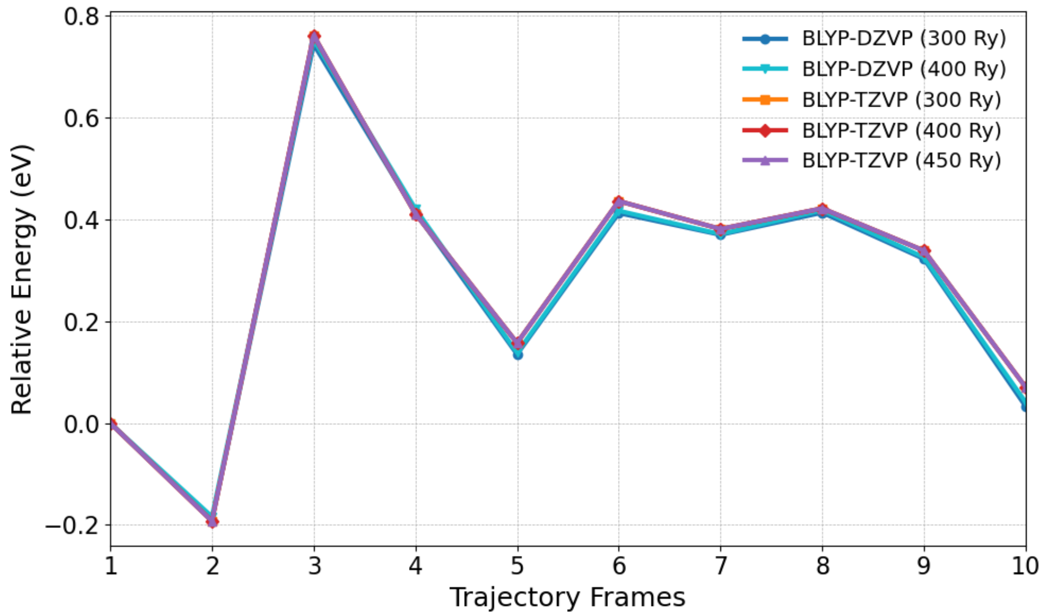

Figure 7: Comparison of single-point energies computed on 10 representative proton-transfer frames using BLYP+D3 with DZVP (300, 400 Ry) and TZVP (300, 400, 450 Ry) settings. All energy differences remain within 0.013 eV.

References

(1) Cochran, W.; Penfold, B. R. The crystal structure of L-glutamine. *Acta Crystallographica* **1952**, *5*, 644–653.

(2) Stephens, A. D.; Qaisrani, M. N.; Ruggiero, M. T.; Díaz Mirón, G.; Morzan, U. N.; González Lebrero, M. C.; Jones, S. T.; Poli, E.; Bond, A. D.; Woodhams, P. J.; others Short hydrogen bonds enhance nonaromatic protein-related fluorescence. *Proceedings of the National Academy of Sciences* **2021**, *118*, e2020389118.
